# Climate change and the threat of deadly dozen

**DOI:** 10.4103/0019-5278.58920

**Published:** 2009-12

**Authors:** Harshal T. Pandve, Kevin Fernandez, Samir A. Singru, P. S. Chawla

**Affiliations:** Department of Community Medicine, Smt. Kashibai Navale Medical College, Narhe, India

Dear Sir,

Climate change is one of the most critical global challenges of our times. Recent events have emphatically demonstrated our growing vulnerability to climate change. The impacts of climate change range from agricultural damage, further endangering food security, to sea-level rise and the accelerated erosion of coastal zones increasing the intensity of natural disasters, species extinction, and spread of vector-borne diseases. This issue is of immense importance for every global citizen. Hence, globally, it requires an initiative against the devastating effects of climate change.[1]

Climate change already contributes to the global burden of disease and this contribution is expected to grow in the future. Direct as well as indirect effects of climate change are alarming to the public health authorities.[2] The health experts from the Wildlife Conservation Society released a report on October 2008 called “Deadly Dozen: Wildlife diseases in the age of climate change.” This report lists 12 pathogens that could spread into new regions as a result of climate change. All have potential impacts to human and wildlife health as well as global economies. The study examined the nuts and bolts of deleterious impacts of climate change on the health of wild animals and the cascading effects on human populations. The “Deadly Dozen” list is illustrative only of the broad range of infectious diseases that threaten humans and animals, it is not a comprehensive one, and subsequent studies may eliminate pathogens from the list of those enabled by climatic factors. The list includes those pathogens that may spread as a result of changing temperatures and precipitation levels. The list includes avian influenza, Babesiosis, cholera, Ebola, intestinal and external parasites, Lyme disease, Plague, Rift Valley Fever, sleeping sickness also known as trypanosomiasis, tuberculosis and yellow fever. It also included harmful algal blooms off global coasts that create toxins that are deadly to both humans and wildlife commonly called “red tides’. It builds upon the recommendations included in a recently published paper titled “Wildlife health as an indicator of climate change,’ which appears in a newly released book, “Global climate change and extreme weather events: Understanding the contributions to infectious disease emergence”, published by the National Academy of Sciences/Institute of Medicine.[3]

To conclude, as per the findings of such reports, climate change already contributes to the global burden of disease and this contribution is expected to grow in the future. Emerging infectious diseases are a major threat to the health and economic stability of the world. As per the report, monitoring of wildlife health provides us with a sensitive and quantitative means of detecting changes in the environment. The best defense, according to the authors of these reports, is a good offense in the form of wildlife monitoring to detect how these diseases are spreading so that health professionals can learn and prepare to mitigate their impact.[3] It is essential for the health policy planners and administrators to consider climate change as a major public health problem in the near future and plan accordingly.
